# Mitochondrial genome sequencing of the monogenean *Heterobothrium okamotoi* isolated from the tiger puffer *Takifugu rubripes* in North China

**DOI:** 10.1080/23802359.2019.1666671

**Published:** 2019-09-18

**Authors:** Ruijun Li, Cheng Zhou, Shigen Ye, Lei Men, Ying Liu, Songzhe Fu

**Affiliations:** a Agriculture and Rural Affairs Ministry of Key Laboratory of Mariculture & Stock Enhancement in North China's Sea, Dalian Key Laboratory of Marine Animal Disease Control and Prevention, College of Fisheries, Dalian Ocean University, Dalian, China;; b College of Marine Technology and Environment, Dalian Ocean University, Dalian, China;; c College of Life Science, Dalian Minzu University, Dalian, China

**Keywords:** Mitogenomics, *Heterobothrium okamotoi*, *Takifugu rubripes*, monogenean

## Abstract

In this study, monogenean *Heterobothium okamotoi* was isolated and identified from the gill of diseased Tiger puffer (*T. rubripes*) at an industrial farm in Liaoning, North China (121.3459 E, 38.9861 N). With the completion of *H. okamotoi* mitochondrial genome sequencing, the full-length mitochondrial genome of *H. okamotoi* was assembled and analyzed. All results indicate that the complete mitochondrial genome of *H. okamotoi* was 14,643 bp. There were 2 rRNAs, 20 tRNAs, and 12 protein-coding genes (PCGs) all located at the heavy (H) strand. Besides, the phylogenetic tree of 19 monogeneans was constructed. The results showed that *H. okamotoi* and *Pseudochauhanea macrorchis* were clustered in a clade. To sum up, our research results would further provide essential data for systematics and evolution study of *H. okamotoi*.

Tiger puffer (*Takifugu rubripes*) belongs to the family Tetraodontidae, genus *Takifugu*. Owing to the fast growth rate and high economic value, *T. rubripes* has already become an important cultured marine fish in China (Gao et al. 2011; Jia et al. 2018). In recent years, with the fast development of tiger puffer factory farming, high-density breeding and low-quality water environment resulted in the frequent occurrence of various diseases, such as bacteria, viral, and parasitic infection (Ishimatsu et al. 2007; Mohi et al. 2010; Li et al. 2018, 2019), to cause severe economic damage and food safety risk. Among these pathogens, the monogenean *Heterobothium okamotoi* was one of the notorious parasites, caused acute death in *T. rubripes* (Ogawa and Inouye 1997a; Ogawa 2016). Some studies reported the ecology, taxonomy, pathology and, immunology, etc. of this parasitic pathogen (Ogawa 1997; Ogawa and Inouye 1997a, 1997b; Wang et al. 1997; Igarashi et al. 2017). However, no report has been made on its complete mitochondrial genome.

In the current study, *H. okamotoi* was isolated and identified from the gill of diseased Tiger puffer (*T. rubripes*) at an industrial farm in Liaoning, North China (121.3459 E, 38.9861 N). Meanwhile, the mitochondrial DNA and specimen of *H. okamotoi* (Number: TRHK01) were preserved and stored in the Dalian Key Laboratory of Marine Animal Disease Control and Prevention, Dalian Ocean University. With the completion of *H. okamotoi* mitochondrial genome sequencing by SC Gene Company (Guangzhou, China) via Illumina MiSeq Next-generation sequencing technique, the full-length mitochondrial genome of *H. okamotoi* was assembled and submitted to the GenBank database (No. MK948930). All analytical results indicate that the complete mitochondrial genome of *H. okamotoi* was 14,643 bp. There were 2 rRNAs (16S rRNA and 12S rRNA), 20 tRNAs (tRNA-Leu, tRNA-Tyr, tRNA-Ser, tRNA-Arg, tRNA-Glu, tRNA-Gln, tRNA-Phe, tRNA-Val, tRNA-Ala, tRNA-Asp, tRNA-Asn, tRNA-Pro, tRNA-Ile, tRNA-Trp, tRNA-Thr, tRNA-Lys, tRNA-Gly, tRNA-Met, tRNA-His and tRNA-Cys), and 12 protein-coding genes (PCGs) all located at the heavy (H) strand. Among these PCGs, 10 genes (*nd6*, *nd5*, *cytb*, *nd4l*, *nd4*, *atp6*, *nd1*, *cox1*, *cox2*, and *cox3*) were with start codon ATG, the rest PCGs *nd2* and *nd3* respectively used the start codon GTG and GTA. Besides, 7 PCGs (*nd6*, *nd5*, *nd4l*, *nd4*, *nd1*, *cox2*, and *cox3*) used the stop codon TAA, and the remaining five genes (*cytb*, *atp6*, *nd2*, *nd3*, and *cox1*) were with the stop codon TAG.

Based on sequences of 12 PCGs (*atp6*, *cox1*, *cox2*, *cox3*, *cytb*, *nd1*, *nd2*, *nd3*, *nd4l*, *nd4*, *nd5*, and *nd6*), the phylogenetic tree of 19 monogeneans was constructed by maximum likelihood method. The results showed that *H. okamotoi* and *Pseudochauhanea macrorchis* were clustered in a clade (Figure 1). Moreover, *H. okamotoi*, *P. macrorchis*, *Polylabris halichoeres,* and *Microcotyle sebastis* grouped, and all just belonged to Polyopisthocotyle (Figure 1).

**Figure 1.**
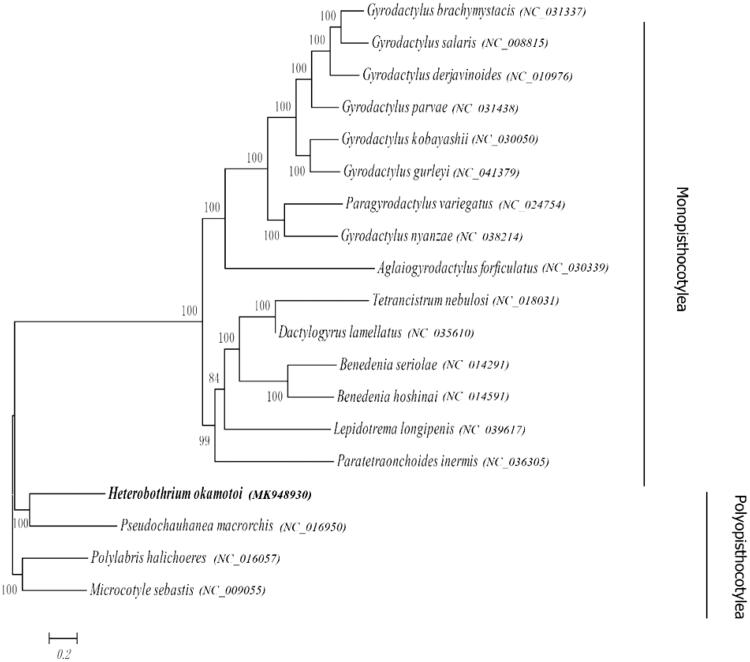
Phylogeny of *Heterobothium okamotoi*. Phylogenetic tree based on nucleotide sequences of PCGs located in the mitogenome. The number of the branches denoted BI posterior probabilities.
